# Sedentary Behaviour, Physical Activity, and Their Associations with Health Outcomes at the Time of Diagnosis in People with Inoperable Lung Cancer

**DOI:** 10.3390/jcm11195870

**Published:** 2022-10-04

**Authors:** Shu Ning Ch’ng, Joanne A. McVeigh, David Manners, Terry Boyle, Carolyn J. Peddle-McIntyre, Rajesh Thomas, Jeanie Leong, Samantha Bowyer, Kirsten Mooney, Leon Straker, Daniel A. Galvão, Vinicius Cavalheri

**Affiliations:** 1 Curtin School of Allied Health, Curtin University, Perth 6845, Australia; 2 enAble Institute, Faculty of Health Sciences, Curtin University, Perth 6845, Australia; 3 St John of God Midland Public and Private Hospitals, Perth 6056, Australia; 4 Australian Centre for Precision Health, Allied Health and Human Performance, University of South Australia Cancer Research Institute, Adelaide 5000, Australia; 5 Exercise Medicine Research Institute, Edith Cowan University, Perth 6027, Australia; 6 Department of Respiratory Medicine, Sir Charles Gairdner Hospital, Perth 6009, Australia; 7 Department of Respiratory Medicine, Royal Perth Hospital, Perth 6000, Australia; 8 Department of Medical Oncology, Sir Charles Gairdner Hospital, Perth 6009, Australia; 9 WA Cancer and Palliative Care Network, North Metropolitan Health Service, Perth 6009, Australia; 10 Allied Health, South Metropolitan Health Service, Perth 6009, Australia

**Keywords:** sedentary behaviour, physical activity, lung cancer

## Abstract

This study aimed to examine sedentary behaviour (SB), physical activity (PA) and their associations with health-related measures at the time of diagnosis in people with inoperable lung cancer. People newly diagnosed with inoperable lung cancer were invited to participate in the study and asked to wear an accelerometer for seven consecutive days. Variables analysed included time spent in SB, light intensity PA (LIPA) and moderate-to-vigorous intensity PA (MVPA). Daily steps were also recorded. Data on symptoms, health-related quality of life (HRQoL), hand grip force, comorbidities and lung function were collected. Of the 120 patients referred to the study, 89 (74%) consented to participate, and SB/PA data were available for 79 (age 71 ± 11 years; 29 females). Participants spent 71% of their waking time in SB, 28% in LIPA and 1% in MVPA. Regression models demonstrated that increased SB was associated with more symptoms of fatigue and dyspnoea (*p* ≤ 0.02 for both), poorer HRQoL (general health and physical component score; *p* ≤ 0.02 for all) and lower hand grip force. For PA variables, higher daily step count was associated with better scores in all health-related measures (*p* < 0.05 for all). LIPA was associated with more health-related outcomes than MVPA. These findings may guide future interventions in this population.

## 1. Introduction

In Australia, lung cancer is the fourth most commonly diagnosed cancer in both males and females, with 12,817 Australians being diagnosed with this malignancy in 2019 [1]. In that same year, over 53% of those with lung cancer in Australia presented with the advanced stage of the disease [1]. Although treatment via lung resection offers the best outcome in people with lung cancer [2], this is not a viable treatment option for people with advanced stage lung cancer or those with early-stage lung cancer who are not “fit enough” to withstand surgery. Patients with inoperable lung cancer have a high symptom burden, poorer health-related quality of life (HRQoL), a more sedentary lifestyle and reduced physical activity (PA) levels when compared to those with other malignancies [3]. The reasons for the high symptom burden and reduced level of PA is multifactorial, but can be partially explained by: (i) the tumour(s) in the lung(s), which disrupt pulmonary mechanics and gas exchange; (ii) underlying chronic lung disease(s), the most common being chronic obstructive pulmonary disease; (iii) the direct effects of lung cancer progression, and (iv) the indirect effects of lung cancer treatment (which vary according to the treatment received) [4,5,6]. Of note, higher level of self-reported leisure-time PA have been associated with lower lung cancer mortality [7].

Current cancer-specific exercise guidelines recommend engaging in at least 90 min of moderate-intensity aerobic exercise per week, in addition to at least 2 days of strength/resistance exercise, per week as needed [8,9,10]. Exercise is a subcategory of PA that is planned, structured, repetitive, and aimed at improving or maintaining one or more components of physical fitness [11]. For people living with cancer, engaging in PA is beneficial for reducing cancer-related fatigue, increasing physical endurance and enhancing overall quality of life [12]. Physical activity is defined as ‘any bodily movement produced by skeletal muscle that results in energy expenditure’ [11]. Sedentary behaviour is defined as ‘any waking behaviour characterised by an energy expenditure ≤1.5 metabolic equivalents (METs), while in a sitting, reclining or lying posture’ [13]. Of note, people with lung cancer have poor PA levels and spend the majority of their waking hours in sedentary behaviour (SB) [3]. 

Both SB and PA can be assessed subjectively and objectively via the use of questionnaires or activity monitors [14], respectively. In studies in people with lung cancer where device-measured SB and PA have been reported [15], substantial methodological heterogeneity has been noticed. These include heterogeneity related to the stage of included participants [16,17], timing of PA measurement along the cancer treatment continuum [4,18], activity monitors used [4,16,18], number of days of activity monitoring [4,16,17,18,19], and the SB and PA variables reported [15]. This heterogeneity limits the transferability of these findings to people with inoperable lung cancer. Further, there is a lack of data on the association between SB/PA and other health-related measures (e.g., symptoms, HRQoL, muscle force, comorbidities, and lung function) at the time of diagnosis (i.e., before treatment commencement) in this population.

The primary aim of this study was to describe SB and PA at the time of diagnosis in people with inoperable lung cancer and investigate the association of SB and PA with symptoms, HRQoL, muscle force, comorbidities, and lung function. Such data could help with the understanding of the potential associations between SB and PA and several health outcomes and could assist in the development of interventions to reduce SB and increase PA during treatment for inoperable lung cancer. We hypothesise that this population will be highly sedentary, and that increased SB and reduced PA will be associated with poorer score/performance in the health outcomes measured.

## 2. Materials and Methods

This study is a cross-sectional analysis (planned a priori) of baseline data collected as part of a longitudinal study aimed at investigating the prognostic value of SB and PA to predict survival in people with inoperable lung cancer. This study was approved by the Human Research Ethics Committee (HREC) at Sir Charles Gairdner Hospital, Perth, Australia (SCGH), Royal Perth Hospital, Perth, Australia (RPH) (Ethics number RGS0000000267 for both) and St John of God Midland Hospital, Perth, Australia (SJoG-Midland) (Ethics number 1293). Reciprocal HREC approval for this study was obtained from Curtin University, Perth, Australia (HRE2017-0683).

### 2.1. Participants

All patients diagnosed with lung cancer seen in the respiratory medicine clinics and/or thoracic oncology clinics at participating tertiary hospitals in Perth (SCGH, RPH, and SJoG-Midland) between February 2018 to December 2020 were screened for potential eligibility. We included adults (aged >18 years) with any type of lung cancer who had been referred for treatments other than lung resection and had not started treatment by the date of the study assessment, with an Eastern Cooperative Oncology Group (ECOG) status [20] ranging from 0 to 2. Patients with brain metastasis, acute illness, on a wheel chair, and/or unable to understand spoken and written English, were excluded.

Research staff discussed the study protocol and procedures over the phone or in person at the respiratory medicine or thoracic oncology clinic and provided potential participants with the participant information and consent form. People who decided to take part in the study were asked to provide informed consent during a face-to-face meeting with research staff, after which the study assessments were conducted.

### 2.2. Measurement/Assessments

Except for accelerometer-measured SB and PA levels, assessments of all other health-related measures occurred during a single 45-min visit prior to the commencement of medical treatment. 

### 2.3. Sedentary Behaviour and Physical Activity

Sedentary behaviour and PA were objectively measured by Actigraph GT9X-Link (Actigraph LLC, Pensacola, FL, USA) accelerometer. Participants were asked to wear the monitor 24 h/day for 7 consecutive days, above the right hip on an elastic belt around the waist. They were also asked to diarise the time they woke in the mornings and the time they went to bed in the evenings. 

### 2.4. Symptoms

Fatigue was assessed using the Functional Assessment of Chronic Illness Therapy (FACIT-F)-fatigue subscale [21], a 13-item questionnaire with each item answered on a five-point rating scale. The questionnaire is based on a seven-day recall period. Scores range between 0 and 52. Numerical ratings are reverse scored when appropriate such that higher scores represent lower fatigue levels. The questionnaire has good reliability and validity based on analyses of people with cancer and rheumatoid arthritis [22]. The minimum important difference (MID) for the FACIT-F is between three to four points [23].

Functional limitation resulting from dyspnoea was assessed by the Modified Medical Research Council (mMRC) dyspnoea scale [24,25] which comprises five statements. Participants select the statement which best reflects their level of limitation in ADLs due to breathlessness. The mMRC dyspnoea scale is a valid method of categorising people with chronic lung disease in terms of their functional disability [26]. Variations of 1 point in the scale are perceived as a clinical change [27].

### 2.5. Health-Related Quality of Life

General HRQoL was assessed using the Medical Outcomes Study Short-Form 36 (SF-36). The SF-36 is a self-complete questionnaire which assesses generic HRQoL [28]. It comprises both a physical (physical component score [SF-36 PCS]) and a mental component (mental component score [SF-36 MCS]). Each component score comprises four subscales. The 36 items of the SF-36 PCS assess physical functioning, physical role functioning, bodily pain, and general health. The SF-36 MCS assesses vitality, social and emotional role functioning, and mental health. Responses to items in each component and each subscale are weighted equally, summed, and transformed to a score that ranges between 0 to 100. Higher scores represent better HRQoL. The SF-36 questionnaire is valid and reliable in people with a range of conditions, including hypertension, chronic heart failure, and chronic obstructive pulmonary disease (COPD) [29,30,31]. The minimum clinically important difference (MCID) for components of the SF-36 PCS and the SF-36 MCS range between three to five points [32].

Lung cancer-specific HRQoL was assessed using the Lung Cancer-Specific Subscale (LCS) of the Functional Assessment of Cancer Therapy—lung scale (FACT-LCS) version 4 and the European Organization for Research and Treatment of Cancer Quality of Life Questionnaire (EORTC QLQ-C30) version 3.

The FACT-LCS is a self-complete nine-item subscale relating to lung cancer-specific symptoms [33]. The total score of the FACT-LCS ranges between 0 and 28. Higher scores represent better HRQoL. This measure was developed for the population of lung cancer patients who are undergoing or have already undergone treatment for lung cancer [33]. The FACT-LCS is responsive to change with good internal consistency and content validity [34]. The MCID for the FACT-LCS ranges between two to three points [35]. 

The EORTC QLQ-C30, which has been validated for use in research [36], is a self-administered questionnaire that assesses the presence of cancer-related symptoms and the degree to which these symptoms affect the individual’s daily activities. For this study, only the two questions relating to global measure of health status were used (EORTC QLQ-C30-GH). These are questions 29 and 30 of the questionnaire [36]. The possible responses for the two questions range from ‘very poor’ (score of one) to ‘excellent’ (score of seven) on a numerical scale. The total score ranges between 2 and 14 and can be transformed to a percentage score. 

### 2.6. Muscle Force

Hand grip force was measured using a hydraulic hand-held dynamometer (Jamar dynamometer; JA Preston Corporation; Jackson, MI, USA). Participants undertook three trials on each hand alternately with a 30 s break between each trial. Peak handgrip force was recorded, with the elbow at 90° of flexion and the forearm and wrist in a neutral position. Measures are expressed in absolute values and as a percentage of the predicted value in a healthy population [37].

### 2.7. Comorbidities

The presence of existing comorbidities and their impact on functional ADLs was assessed using the Self-Administered Comorbidities Questionnaire (SCQ), a reliable [38] self-administered questionnaire. The questionnaire consists of a series of closed-ended questions such as ‘Do you have any of the following problems?’ in relation to 12 medical conditions specified within the questionnaire. Individuals are also given the option of completing an additional open-ended question that requires them to write in any ‘other medical problems’. A maximum of three points are scored for each medical condition—one point for the presence of the medical condition, one additional point if treatment was being received for the condition, and one final point if the medical condition caused limitations in functional activities. The score for the number of comorbidities ranges between 0 and 15 [38]. The total score of the SCQ ranges between 0 and 45.

### 2.8. Clinical Covariates

Data relating to lung cancer diagnosis (i.e., stage and test results) and treatment, as well as results of the most recent spirometry [i.e., forced expiratory volume in one second (FEV_1_) and forced vital capacity (FVC)] and results of the most recent blood test (e.g., lymphocyte, neutrophil, and platelet count), were extracted from medical records. 

### 2.9. Data Management and Analysis

Data collected with the Actigraph GT9X-Link were downloaded and processed in SAS (version 9.3, SAS Institute, Cary, NC, USA). A minimum of 2 days with at least 10 h of wear time was needed to be included in the analysis as this population is known for being highly sedentary. The Actigraph was programmed to record raw data at a frequency of 30 Hz. Using 1-min epoch data of counts/min of the vertical axis, the proportion of time spent in 3 domains was determined: (i) sedentary time (<100 counts/min [39]); (ii) light-intensity PA (LIPA) (≥100 and <1951 counts/min [40]); and (iii) moderate-to-vigorous intensity PA (MVPA) (≥1951 counts/min [40]). The usual sedentary bout duration (UBD) was calculated for each participant, from the midpoint of each participant’s sedentary accumulation curve, fitted by non-linear regression as described by Chastin and Granat (2009) [41]. Bouts of sedentary time lasting longer than 60 min and time spent in bouts of sedentary behaviour lasting between 30 to 60 min were reported as descriptors only (not used in the regression models to minimize multiple testing). To account for extraneous steps, the average number of steps per day as counted by the Actigraph were filtered to only include epochs of more than 100 counts per minute.

Data analysis was conducted using the Statistical Package for Social Sciences (SPSS) version 26.0 (Chicago, IL, USA). The distribution of continuous data was determined using histograms and the Shapiro–Wilk test. Data that had a normal distribution were expressed as mean and standard deviation (SD). Data that did not have a normal distribution were expressed as median and interquartile range (IQR). Linear correlations between SB (time sent sedentary and UBD)/PA variables and symptoms, HRQoL, muscle force, comorbidities, and lung function were tested using either Pearson’s or Spearman’s correlation coefficients. Regression models (linear or binary logistic) were used to determine the associations of SB/PA variables and symptoms, HRQoL, muscle force, comorbidities, and lung function. The score of the mMRC scale was dichotomised (i.e., ≤1 [no functional limitation resulting from dyspnoea] or ≥2 [functional limitation resulting from dyspnoea]) so that a binary logistic regression was run. All regression models were adjusted for age, gender, body mass index (BMI), lung cancer stage (i.e., early vs. advanced), neutrophil to lymphocyte ratio (NLR), and waking wear time (min/day). Results are presented as either beta coefficients (β) or odds ratio (OR) with their respective 95% confidence intervals (CI). For all analyses, *p*-Values of <0.05 were considered statistically significant. 

## 3. Results

### 3.1. Participant Characteristics and Compliance with Actigraphy

**Figure 1:**
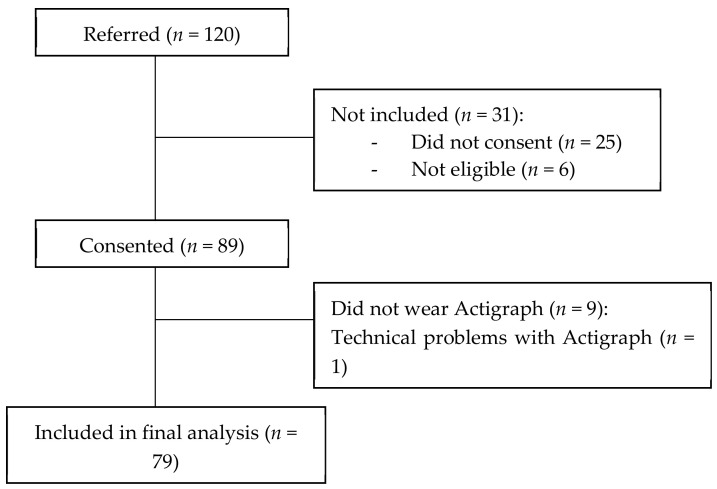
Flow of participants throughout this study.

One hundred and twenty potential participants were referred to our study. Eighty-nine consented to participate (Figure 1). All 89 Actigraphs were returned. Nine participants (10%) did not wear the Actigraph: eight due to personal reasons and one due to hospital admission. One participant returned the Actigraph, but their data could not be analysed due to technical problems. Eighty-eight of the 89 participants were seen face-to-face. One participant lived in a rural area in Western Australia and was not seen face-to-face. As the participant agreed to have the Actigraph and questionnaires mailed to him/her, he/she was included in the study. However, data on hand grip force was not able to be collected for this participant. The characteristics of the included participants are described in Table 1.

### 3.2. Sedentary Behaviour and Physical Activity

Of the 79 participants whose data were analysed, 59 (75%) contributed seven valid days, 14 (18%) contributed six valid days, and six (7%) contributed five or fewer valid days. Table 2 presents a summary of the data collected using the Actigraph. The mean daily wear time was 14.8 h. Participants spent 71% of their waking time in SB, 28% in LIPA, and 1% in MVPA.

### 3.3. Correlations

Table 3 presents correlations between variables related to SB/PA with symptoms, HRQoL, muscle force, comorbidities, and lung function. In general, weak to moderate correlations were observed. The highest r-value detected was between daily step count and SF-36 PCS (r = 0.58). The SF-36 MCS was not correlated with any variable of SB/PA. 

Both SB variables (i.e., time spent in SB and UBD) presented a significant inverse correlation with fatigue scores (FACIT-F; higher scores represent lower fatigue levels), physical component of the general HRQoL questionnaire (SF-36 PCS), cancer-specific HRQoL (EORTC QLQ-C30-GH), and hand grip force (%predicted) (r-values between −0.38 and −0.22). No significant correlations were observed between either SB variables with the mental component of HRQoL (SF36-MCS) nor lung cancer-specific HRQoL (FACT-LCS). Increased time spent in SB and increased UBD were significantly associated with increased odds of having functional limitation resulting from dyspnoea (mMRC scale) (unadjusted OR [95% CI] 1.46 [1.08, 1.96] and 1.09 [1.03, 1.17], respectively).

All PA variables (i.e., time spent in LIPA, MVPA and daily step count) presented a direct correlation with the FACIT-F, SF-36 PCS, EORTC QLQ-C30 scores, hand grip force (%predicted) (r-values between 0.29 and 0.58), and an inverse correlation with the SCQ score (r-values between −0.37 and −0.25). Time spent in LIPA and daily step count also presented a direct correlation with the FACT-LCS (r-values = 0.26 and 0.30, respectively). Increased time spent in LIPA, MVPA, and increased daily step count were associated with decreased odds of having functional limitation resulting from dyspnoea (mMRC scale) (OR [95% CI] 0.48 [0.31, 0.74], 0.94 [0.88, 0.99], and 0.84 [0.76, 0.92], respectively). Lung function (i.e., FEV_1_ (%predicted)) was only correlated with MVPA (r = 0.28).

### 3.4. Summary of the Adjusted Regression Models

Table 4 presents a summary of the results of the regression models adjusted for age, gender, BMI, lung cancer stage (i.e., early vs. advanced), NLR, and waking wear time (min/day). The SF-36 MCS was not included in the regression analyses due to lack of correlation with SB and PA variables. Both the SF-36 PCS and functional limitation resulting from dyspnoea (mMRC score) were associated with all SB and PA variables.

Every h/day increment in time spent in SB was associated with a 1.65-point reduction in FACIT-F (i.e., greater fatigue), increased odds of having functional limitation resulting from dyspnoea (mMRC scale) (OR [95% CI] 2.3 [1.45, 3.65]), a 2-point reduction in SF-36 PCS, a 3.6-point reduction in EORTC QLQ-C30-GH, and a 2.55% reduction in hand grip force. Usual bout duration was not associated with HRQoL, comorbidities, or lung function. Every minute increment in UBD was associated with a 0.33-point reduction in FACIT-F (i.e., greater fatigue), increased odds of having functional limitation resulting from dyspnoea (mMRC scale) (OR [95% CI] 1.12 [1.04, 1.20]), a 0.33-point reduction in SF- 36 PCS, a 0.52-point reduction in EORTC QLQ-C30-GH and a 0.45% reduction in hand grip force.

Time spent in LIPA was not associated with comorbidities (SCQ total score) or lung function (FEV_1_). Every h/day increment in time spent in LIPA was associated with a 3.7- point increment in FACIT-F (i.e., less fatigue), decreased odds of having functional limitation resulting from dyspnoea (mMRC scale) (OR [95% CI] 0.44 [0.27, 0.70]), a 3.6- point increment in SF-36 PCS, a 1 point increment in the FACT-LCS, a 6.4-point increment in the EORTC QLQ-C30-GH, and a 3.6% increase in hand grip force. 

Time spent in MVPA was not associated with FACT-LCS or comorbidities (SCQ total score). Every h/day increment in time spent in MVPA was associated with a 0.24-point increment in FACIT-F (i.e., less fatigue), decreased odds of having functional limitation resulting from dyspnoea (mMRC scale) (OR [95% CI] 0.92 [0.85, 0.99]), a 0.21 point increment in the SF-36 PCS, a 0.51-point increase in the EORTC QLQ-C30-GH, and a 0.38% increment in hand grip force. 

Daily step count (500 steps increments) was associated with all outcomes. Every 500- step increment was associated with a 0.86-point increase in FACIT-F (i.e., less fatigue), decreased odds of having functional limitation resulting from dyspnoea (mMRC scale) (OR [95% CI] 0.81 [0.73, 0.91]), a 0.85-point increase in SF-36 PCS, a 0.26-point increase in FACT-LCS, a 1.69-point increase in EORTC QLQ-C30-GH, a 0.64% increase in hand grip force, a 0.15-point reduction in the SCQ total score, and a 0.81% increase in FEV_1_.

## 4. Discussion

This study specifically aimed at collecting device-measured SB and PA data prior to treatment commencement in people with inoperable lung cancer. Our participants spent 71% of their waking time in SB, 28% in LIPA and 1% in MVPA. Regression models demonstrated that increased SB was associated with more symptoms of fatigue and dyspnoea, poorer HRQoL (general health and physical component score) and lower hand grip force. For PA variables: (i) higher daily step count was associated with better scores in all health-related measures; and (ii) LIPA was associated with more health-related outcomes than MVPA. Of note, associations between SB and PA with comorbidities and lung function were limited.

At the time of diagnosis, people with inoperable lung cancer who took part in this study were highly sedentary (71% of waking hours) and spent minimal time in MVPA (only 1% of waking hours). This is concerning as these people are starting the lung cancer journey with a poor SB/PA profile position. A systematic review [42] demonstrated that regular participation in leisure-time PA pre-lung cancer diagnosis was associated with reduced hazards of mortality among lung cancer patients. The association between SB/PA immediately following a lung cancer diagnosis with survival has not yet been investigated, but our data may help support the idea that early management of inoperable lung cancer should include investigation of SB/PA and interventions aimed at improving the SB/PA profile in this population. 

A previous study in a different lung cancer population (people following surgery for NSCLC) [43] demonstrated that participants spent 68% of their waking time in SB, 21% in LIPA, 11% in MVPA and performed 8863 steps/day. The larger proportion of waking hours being spent in MVPA in that study as well as the greater mean daily step count may be due to those participants: (i) having early-stage lung cancer, (ii) having undergone curative-intent treatment, and (iii) because the participants had worn monitors other than the Actigraph. They used the SenseWear armband, worn on the arm, to measure MVPA. This device may detect activities performed with the upper limbs only, which are not detected with the Actigraph (worn on the waist in the current study). To measure step count, that previous study used the StepWatch, worn on the ankle, which may provide a more accurate reading of step count than the Actigraph. Conversely, the mean step count in the current study is greater than the median step count reported at baseline in a previous randomised controlled trial (between 2860 and 3195 steps/day) in people with inoperable lung cancer [4]. Of note, in that previous trial, step count was measured via the SenseWear armband, which is worn on the arm and known for underestimating step count in people with chronic lung disease [44]. Further, that trial included people who commenced treatment ≤4 weeks prior to recruitment. Therefore, the low step count can also be due to the negative effects of treatment (chemo/radiation therapy) on PA.

Even though only people with ECOG status between 0 and 2 (i.e., considered to be up and about more than 50% of waking hours) were included in the current study, the mean time per day spent in SB was over 10 h (630.3 ± 111.2 min/day; 71% of waking hours). This demonstrates that people who are deemed able to be up and about more than 50% of waking hours may not spend more than 50% of waking hours up and about. This high mean time per day spent in SB is similar to that reported in survivors of lung cancer (9.8 h/day; 69.4% of waking hours) [16] and prostate cancer (10 h/day; 71.4% of waking hours) [45], and more than that reported in community-dwelling older women (8.7 h/day; 67.7% of waking hours) and survivors of breast cancer, colon cancer and non-Hodgkin lymphoma (between 8 and 9 h/day; or between 56.5% and 65.8% of waking hours) [46,47,48,49]. This is concerning as prolonged sedentary time is associated with adverse health outcomes in people with cancer [50,51,52]. These correlations with adverse outcomes are also demonstrated in the current study. We showed that increased SB was associated with more fatigue and functional limitation resulting from dyspnoea, worse HRQoL (physical component) and poorer muscle force. Therefore, future studies should explore ways of reducing prolonged sedentary time and breaking up sedentary time in people with inoperable lung cancer and investigate if a positive change in SB leads to improvements in health outcomes.

In comparison to SB, we observed more and stronger associations between daily step count and LIPA with health measures. In fact, daily step count (in increments of 500 steps) was associated with all health outcomes measured. Clinically, step count is a variable that is easy to measure and may provide useful information regarding general health. Increments of 500 steps, which is a discrete dose, could easily be used to aid interventions that include PA goal setting. Of note, previous study in people with COPD, a common comorbidity in those with lung cancer, demonstrated that people who improve >600 steps following pulmonary rehabilitation have a lower risk of hospital admission [42]. The associations between time spent in LIPA with health outcomes demonstrated in the current study corroborate findings from a previous study in 127 survivors of NSCLC [16]. That study demonstrated time spent in LIPA to be positively associated with HRQoL, fatigue as well as physical and functional well-being. It is worth mentioning that in the current study, every h/day increment in time spent in LIPA was associated with clinically important differences in fatigue (3.7 points in the FACIT-F; MID = 3 to 4 points), and in SF- 36 PCS (3.6-point increment; MCID = 3 to 5). The current study has also shown weaker associations between time spent in MVPA with fatigue, functional limitation resulting from dyspnoea, physical components of HRQoL and FEV_1_. Time spent in LIPA was only not associated with comorbidities and lung function (FEV_1_). The limited association between SB/PA and comorbidities in our participants contrast the findings of previous studies in people with cancer [53,54] and in people with COPD [55]. However, in these previous studies participants did not have lung cancer, were substantially younger (mean age between 48 and 53) than in the current study, and SB/PA levels were either self-reported or estimated using a questionnaire. Further, comorbidity data were collected in previous studies as either self-reported (answering to questions such as: “Have you ever been diagnosed with…?”) or via evaluating the severity of comorbidities with the Charlson Comorbidity Index. As a result, comparison between our findings and previous findings does not seem to be appropriate.

### Strengths and Limitations

This was the first study to report on device-measured time spent in SB and PA specifically prior to treatment commencement in people with inoperable lung cancer. We had a good recruitment rate (74%) compared to previous studies in this population [4,56]. We acknowledge that this was a cross-sectional study, therefore causality of associations cannot be determined. The waist-worn accelerometer can misclassify activities in standing as time spent in SB (rather than LIPA), resulting in overestimation of time spent in SB [57]. To address this issue, we recommend the use of a thigh-worn activity monitor in future studies. As our study included only participants with better functional status (i.e., ECOG ≤ 2), our results may not be generalisable to people with worse functional status.

## 5. Conclusions

In people newly diagnosed with inoperable lung cancer, both device-measured SB and PA were associated with several health outcomes. Compared to SB, we found more associations between daily step count and LIPA with fatigue, functional limitation resulting from dyspnoea, physical components of HRQoL and muscle force. Associations between SB and PA with comorbidities and lung function were limited. In this population, an initial and continuing intervention goal of reducing SB by replacing it with steps performed at LIPA has the potential to lead to clinically meaningful change in fatigue and HRQoL and would be more achievable than a goal of replacing SB with MVPA. Future research should (i) explore ways of increasing LIPA in people newly diagnosed with inoperable lung cancer, and (ii) investigate if improving PA levels in these people leads to improvements in health outcomes.

## Figures and Tables

**Table 1:** Characteristics of the 79 people with inoperable lung cancer included in the study.

Variable	*n*	Value ^1^
Age, *year*	79	70.5 ± 11.1
Sex, *female (%)/male (%)*	79	29 (37%)/50 (63%)
BMI, *kg·m^−2^*	79	25.1 [22.0 to 30.5]
Employment status, *n (%)*	79	
Disability		8 (10%)
Retired		46 (58%)
Part time		7 (9%)
Full time		11 (44%)
Homemaker		1 (1%)
Temporarily unemployed		6 (8%)
Smoking status, *n (%)*	79	
Current smoker		19 (24%)
Ex-smoker		53 (67%)
Never smoked		7 (9%)
Smoking, *pack·years*	72	40 [25 to 62]
Type of cancer, *n (%)*	79	
*Non-small cell lung cancer*		
Adenocarcinoma		44 (56%)
Squamous cell carcinoma		24 (30%)
Large cell		2 (3%)
Adenosquamous carcinoma		1 (1.3%)
*Small cell lung cancer*		5 (6%)
*Poorly differentiated carcinoma/unknown*		1 (1.3%)/2 (2.6%)
NSCLC/SCLC stage, *n (%)*	79	
Early (I to IIIA)/limited		32 (41%)
Advanced (IIIB to IV)/extensive		47 (59%)
ECOG status, n (%)	79	
0 / 1 / 2		26 (33%)/40 (51%)/13 (17%)
COPD, *n (%)*	79	
Yes		33 (42%)
No		41 (52%)
Unsure		5 (7%)
FEV_1_, *L*	63	1.9 ± 0.7
FEV_1_, *%pred*	63	72.6 ± 25.1
FVC, *L*	63	3.0 ± 1.0
FVC, *%pred*	63	85.3 ± 22.1
FEV_1_/FVC, *%*	63	63.1 ± 15.9
HRQoL	79	
FACT-LCS (0 to 28)		20 ± 5
SF-36 PCS (0 to 100)		44 ± 11
SF-36 MCS (0 to 100)		41 ± 10
EORTC QLQ-C30-GH (0 to 100)		61 ± 23
Symptoms	79	
Fatigue (FACIT-F)		38 ± 12
Dyspnoea (mMRC), *n (%)*		
0 / 1 / 2 / 3 / 4		17 (22%)/38 (48%)/16 (20%)/7 (9%)/1 (1%)
Comorbidities	79	
SCQ Number of comorbidities		5 ± 4
SCQ Total score		10 ± 4
SCQ comorb. affecting activities		2 ± 1
Muscle force	78 **	
Hand grip force, *Nm*		30 ± 9
Hand grip force, *%pred*		94 ± 24
Blood cell count	78 **	
Neutrophils, ×*10^9^/L*		7 ± 4
Platelets, ×*10^9^/L*		312 ± 136
Lymphocytes, ×*10^9^/L*		2 ± 1
NLR		3.2 [2.4 to 5.4]

^1^ Data are presented as mean ± standard deviation or median (interquartile range), unless otherwise stated. Lung function data (i.e., FEV_1_ and FVC) were only available in 63 participants. ** Not assessed for one participant who resided in a rural area in Western Australia and was not seen face-to face. Abbreviations: BMI: Body mass index; ECOG: Eastern Cooperative Oncology Group; FACIT-F: Functional Assessment of Chronic Illness Therapy–fatigue subscale; FACT-LCS: Lung Cancer Subscale of the Functional Assessment of Cancer Therapy; FEV_1_: Forced expiratory volume in one second; FVC: Forced vital capacity; L: Litres; mMRC: Modified Medical Research Council dyspnoea scale; NLR: Neutrophil-to-lymphocyte ratio; NSCLC: Non-small cell lung cancer; PA: Physical activity; SCLC: Small cell lung cancer; SCQ: Self-Administered Comorbidities Questionnaire; SF-36 MCS: Medical Outcomes Study Short Form 36–Mental Component Score; SF-36 PCS: Medical Outcomes Study Short-Form 36–Physical Component Score.

**Table 2:** Sedentary behaviour and physical activity among people with inoperable lung cancer in Western Australia, as measured via Actigraph GT9X-Link accelerometer (*n* = 79).

Variable	Value ^1^
Number of valid days	7 [6 to 7]
Waking wear time, min/day	887.4 ± 86.3
Time spent sedentary, min/day	630.3 ± 111.2
Time spent in LIPA, min/day	245.6 ± 88.0
Time spent in MVPA, min/day	4.6 [1 to 14.6]
Daily step count, steps/day	7768 ± 3758
UBD (min)	18.1 [13.4 to 23.8]
Time spent in sedentary bouts 30 to <60 min, min/day	136.7 [101.5 to 184.7]
Time spent in sedentary bouts ≥60 min, min/day	79.5 [31.4 to 123.4]
Number of sedentary bouts of >60 min, bouts/day	1 [0.3 to 1.4]

^1^ Data are presented as mean ± standard deviation or median (interquartile range). Abbreviations: LIPA: light intensity physical activity; MVPA: moderate-to-vigorous intensity physical activity; UBD: ‘usual’ sedentary bout duration.

**Table 3:** Correlations (either r-values or unadjusted odds ratio) between sedentary behaviour/physical activity variables and symptoms (FACIT-F and mMRC), HRQoL, muscle force, comorbidities, and lung function.

Variables	FACIT-F ^†^ (r-Value)	mMRC (OR [95% CI])	SF-36 PCS (r-Value)	SF-36 MCS (r-Value)	FACT-LCS (r-Value)	EORTC QLQ-C30-GH (r-Value)	Hand Grip Force (%pred) (r-Value)	SCQ Total Score (r-Value)	FEV_1_ % pred) (r-Value)
Sedentary behaviour									
Time spent in sedentary behaviour (h/day)	−0.22 *	1.46 (1.08, 1.96) **	−0.39 **	−0.06	−0.12	−0.28 *	−0.38 **	0.21	0.04
UBD (min)	−0.20 *	1.09 (1.03, 1.17) **	−0.35 **	−0.07	−0.02	−0.23 *	−0.37 **	0.21	−0.04
**Physical activity**									
Time spent in light intensity physical activity (h/day)	0.40 **	0.48 (0.31, 0.74) **	0.50 **	0.13	0.26 *	0.43 **	0.30 **	−0.25 *	0.02
Time spent in moderate-to-vigorous physical activity (min/day)	0.29 **	0.94 (0.88, 0.99) *	0.40 **	0.04	0.15	0.34 **	0.34 **	−0.37 **	0.28 *
Daily step count (steps/day)	0.46 **	0.84 (0.76, 0.92) **	0.58 *	0.10	0.30 *	0.46 **	0.34 **	−0.35 **	0.06

* *p* < 0.05; ** *p* ≤ 0.01. ^†^ FACIT-F: higher scores represent lower fatigue levels. Abbreviations: FACIT-F: Functional Assessment of Chronic Illness Therapy–fatigue subscale; FACT-LCS: Lung Cancer Subscale of the Functional Assessment of Cancer Therapy; FEV_1_: Forced expiratory volume in one second; EORTC QLQ-C30-GH: The European Organization for Research and Treatment of Cancer Quality of Life Questionnaire version 3–general health component; mMRC: Modified Medical Research Council dyspnoea scale; OR: Odds ratio; SCQ: Self-Administered Comorbidities Questionnaire; SF-36 MCS: Medical Outcomes Study Short Form 36–Mental Component Score; SF-36 PCS: Medical Outcomes Study Short Form-36–Physical Component Score; UBD: ‘Usual’ sedentary bout duration.

**Table 4:** Associations between sedentary behaviour/physical activity variables and symptoms (FACIT-F and merch), HRQoL, muscle force, comorbidities, and lung function. Values adjusted for age, gender, BMI, lung cancer stage, NLR, and waking wear time (min/day).

Variables	FACIT-F * (β)	mMRC (*OR*)	SF-36 PCS (β)	FACT-LCS (β)	EORTC QLQ-C30-GH (β)	Hand Grip Force (%pred) (β)	SCQ Total Score (β)	FEV_1_ (%pred) (β)
Sedentary behaviour								
Time spent in sedentary behaviour (h/day)	−1.65 (−3.03, −0.27) *p* = 0.02	2.3 (1.45, 3.65) *p* < 0.001	−1.97 (−3.17, −0.77) *p* < 0.01	−0.40 (−0.97, 0.16) *p* = 0.17	−3.63 (−6.26, −1.00) *p* < 0.01	−2.55 (−4.98, −0.11) *p* = 0.04	0.44 (−0.05, 0.93) *p* = 0.08	−1.57 (−4.42, 1.29) *p* = 0.28
UBD (min)	−0.33 (−0.55, −0.10) *p* < 0.01	1.12 (1.04, 1.20) *p* < 0.01	−0.33 (−0.53, −0.13) *p* < 0.01	−0.04 (−0.13, 0.06) *p* = 0.43	−0.52 (−0.97, −0.08) *p* = 0.02	−0.45 (−0.86, −0.04) *p* = 0.03	0.06 (−0.03, 0.14) *p* = 0.17	−0.34 (−0.80, 0.13) *p* = 0.16
**Physical activity**								
Time spent in light intensity physical activity (h/day)	3.73 (2.13, 5.33) *p* < 0.01	0.44 (0.27, 0.71) *p* < 0.01	3.60 (2.20, 5.00) *p* < 0.01	1.00 (0.31, 1.68) *p* < 0.01	6.44 (3.28, 9.61) *p* < 0.01	3.61 (0.55, 6.66) *p* = 0.02	−0.59 (−1.21, 0.02) *p* = 0.06	3.46 (−0.38, 7.30) *p* = 0.08
Time spent in moderate-to-vigorous physical activity (min/day)	0.24 (0.08, 0.39) *p* < 0.01	0.92 (0.85, 0.99) *p* = 0.02	0.21 (0.07, 0.35) *p* < 0.01	0.05 (−0.02, 0.12) *p* = 0.13	0.51 (0.21, 0.81) *p* < 0.01	0.09 (−0.20, 0.38) *p* = 0.55	−0.05 (−0.11, 0.01) *p* = 0.10	0.38 (0.03, 0.72) *p* = 0.03
Accelerometer filtered steps (500 steps increments)	0.86 (0.55, 1.17) *p* < 0.01	0.81 (0.72, 0.91) *p* < 0.001	0.85 (0.58, 1.11) *p* < 0.01	0.26 (0.12, 0.39) *p* < 0.01	1.69 (1.10, 2.29) *p* < 0.01	0.64 (0.01, 1.26) *p* = 0.045	−0.15 (−0.28, −0.03) *p* = 0.01	0.81 (0.02, 1.59) *p* = 0.04

Data are presented as β (95% confidence interval) for SF-36 PCS, FACT-LCS, EORTC QLQ-C30-GH, FACIT-F, SCQ total score, Hand grip force (%predicted) and lung function percentage of predicted (FEV_1_ (%predicted)). Data are presented as odds ratio (OR) (95% confidence interval) for mMRC where the odds of having a ‘good’ mMRC score (i.e 0 to 1) are reported. * FACIT-F: higher scores represent lower fatigue levels Abbreviations: BMI: Body mass index; FACIT-F: Functional Assessment of Chronic Illness Therapy–fatigue subscale; FACT-LCS: Lung Cancer Subscale of the Functional Assessment of Cancer Therapy; FEV_1_: Forced expiratory volume in one second; mMRC: Modified Medical Research Council dyspnoea scale; EORTC QLQ-C30-GH: The European Organization for Research and Treatment of Cancer Quality of Life Questionnaire version 3–general health component; SCQ: Self-Administered Comorbidities Questionnaire; SF-36 PCS: Medical Outcomes Study Short Form-36–Physical Component Score; UBD: Usual sedentary bout duration.

## Data Availability

Please contact authors to obtain any data from the study.
